# Expanding the geographic and host range of moss-nostocacean symbioses in central Europe

**DOI:** 10.1007/s00442-026-05990-7

**Published:** 2026-10-23

**Authors:** Martin Feulner, Ulla Spieleder, Frederic J. Pascual Dose, Jona Motzkus, Daniel C. Thomas

**Affiliations:** 1 Plant Systematics, University of Bayreuth, Universitätsstraße 30, 95447 Bayreuth, Germany; 2 Bayreuth Center of Ecology and Environmental Research (BayCEER), University of Bayreuth, Dr. Hans-Frisch-Str 1-3, 95448 Bayreuth, Germany; 3 Program for Ecological Engineering (M.Sc.), Technical University of Munich, Arcisstraße 21, 80333 Munich, Germany; 4 Department of Ecological Microbiology, Bayreuth Center of Ecology and Environmental Research (BayCEER), University of Bayreuth, Dr. Hans-Frisch-Str 1-3, 95448 Bayreuth, Germany; 5 Program for Biodiversity: Ecology, Evolution, and Conservation (M.Sc.), University of Göttingen, Wilhelmsplatz 1 (Aula), 37073 Göttingen, Germany

**Keywords:** Nostocaceae, Hypnales, Orthotrichaceae, Zygodon, Neckeraceae, Amblystegiaceae, Endophyte, Cyanobacteria

## Abstract

Since their re-discovery, Moss-cyanobacterial symbioses are of increasing interest, especially *Nostoc*-feather moss symbioses. Efforts to document *Nostoc*-feather moss symbioses have historically been centered in Scandinavia and the North American Boreal forest zone. Herein we report the results of several opportunistic surveys investigating the occurrence of Nostocaceae-moss symbioses in the region of Franconia (Germany), from *Hylocomium splendens* of the forest floor and four moss species (*Orthotrichum lyellii, Zygodon dentatus, Homalia trichomanoides, Pseudoamblystegium subtile*) located on three tree species (*Fagus sylvatica*, *Fraxinus excelsior*, and *Sorbus schuwerkiorum*). Using culture- and microscopy-based methods, we confirm the occurrence of such symbioses in our region. Four new moss host species for the symbiosis are documented, from the epiphytic family Orthotrichaceae (*Orthotrichum* sp. and *Zygodon* sp.), and also Neckeraceae (*Homalia trichomanoides*) and Amblystegiaceae (*Pseudoambylstegium subtile*). This further extends the range of known moss hosts for *Nostoc* and *Nostoc*-related organisms outside the traditional feather moss (Hypnales) and *Sphagnum* clades. 16S rRNA gene sequence alignments placed the moss-associated *Nostoc* and *Nostoc-*related organisms throughout our Nostocaceae phylogeny, with no obvious single symbiotic clade. We note the presence of multiple alternate *nifH* genes within the genomes of our cultured *Nostoc* and *Nostoc*-related organisms, possibly enabling diazotrophy under variable ecological conditions. We also present evidence for possible endophytic colonization by cyanobacteria of stems of mosses.

**Supplementary Information:**

The online version contains supplementary material available at https://doi.org/10.1007/s00442-026-05990-7.

## Introduction

Close associations between *Nostoc* cyanobacteria and mosses have been noted by naturalists for over a century. Among the first described species of *Nostoc* (sensu latu, *Nostoc* Vaucher ex Bornet and Flahault 1886) were specimens collected from mosses (*Nostoc muscorum* Agardh ex Bornet et Flahault 1888, syn. *Desmonostoc muscorum* (Hrouzek et al. 2013). Researchers throughout the twentieth century continued to note frequent associations of *Nostoc* and closely related organisms (hereafter referred together as “*Nostoc-*like organisms”) and mosses (division Bryophyta) (Meeks 1990). The potential importance of these associations at larger scale in temperate and arctic terrestrial ecosystems was predicted and quantified in multiple early studies (Dodds et al. 1995). While all mosses are known to host cyanobacterial communities (Groß et al. 2024; Rousk 2022), especially *Sphagnum* mosses (Rousk et al. 2015), interest in bryophyte-nostocacean associations grew rapidly following the documentation of a widespread symbiosis between a *Nostoc-*like organism and a feather moss *Pleurozium schreberi* in boreal forests (DeLuca et al. 2002). The super-abundance of *Pleurozium* and other feather mosses (order Hypnales) in the large northern forest zones implied immense importance for global-scale N and C budgets. Since these findings, research on feather moss/nostocacean symbioses has accelerated and studies have employed increasingly sophisticated means of detection and quantification of nitrogen-fixation and nutrient transfer (Alvarenga et al. 2024; Arróniz-Crespo et al. 2022; Bay et al. 2013; Stuart et al. 2020).

Moss-cyanobacterial mutualisms appear to strongly contribute to the primary production of boreal forests and therefore contributes to ecosystem services (timber production) and sustainability (reduced fertilizer inputs). Through associations with nitrogen-fixing microorganisms, mosses can provide large inputs of nitrogen for ecosystems. In boreal forests, up to 50% of the nitrogen input may come from cyanobacteria associated with mosses (Rousk and Michelsen 2017). In peatland cyanobacteria can contribute more than 35% of the nitrogen required by mosses like *Sphagnum*, helping maintain the productivity and stability of these carbon-rich environments (Berg et al. 2013). In harsher climates, such as tundra regions (reviewed by Lindow et al. (2013)) or tree-canopy microclimates (Lindo and Whiteley 2011), cyanobacteria associated with mosses can account for the vast majority of the nitrogen-fixation necessary for observed rates of net primary production. Variation in the community of nitrogen-fixing bacteria – and especially cyanobacteria – appears to be explained by both geography (Groß et al. 2024) and the host specificity (Ininbergs et al. 2011).

Following classical, broad definitions of symbiosis as any long-term, physically-proximal biotic interaction (Tipton et al. 2019), symbioses between feather mosses and *Nostoc*-like organisms are generally considered to be “loose”, mutually-facultative or commensal exosymbioses. *Nostoc*-like organisms appear to prefer to epiphytically inhabit the spaces in protected leaflet-axils along moss caulids (“stems”), or in incurved regions of leaflet-tissue and other protected areas (DeLuca et al. 2002; Solheim et al. 2004), suggesting at least a structural benefit for *Nostoc*-like organisms when associating with moss hosts. Indeed, the main advantage for the cyanobacterium may be a buffered environment on the moss, as the hygroscopic nature of mosses may create sheltered microenvironments even during times of high radiative exposure (Maeng et al. 2025) or drought (Li et al. 2024) for their resident microbiome.

Benefits from the moss-nostocacean symbioses for the moss host are presumed, as chemo-attraction and induction of mobile hormogonia phase by feather mosses in *Nostoc*-like symbionts have been clearly documented (Bay et al. 2013), at an energetic cost to the host moss (Sprent and Meeks 2013). Nutrient exchanges are hypothesized, in the form of fixed, organic nitrogen from heterocystous *Nostoc*-like colonies to the host plant (Arróniz-Crespo et al. 2022; Bay et al. 2013), perhaps in exchange for organic carbon- and sulfur-containing materials from the moss host, though the latter has only recently been directly reported (Stuart et al. 2020). Symbioses vary seasonally and daily (Warshan et al. 2016) in their levels of nitrogen-fixing activity and microbial abundance, due to climatic constraints on microbial metabolism (Gentili et al. 2005) and N-availability at both host (Renaudin et al. 2022) and ecosystem scales (Cusack et al. 2009; Zheng et al. 2019).

**Fig. 1:**
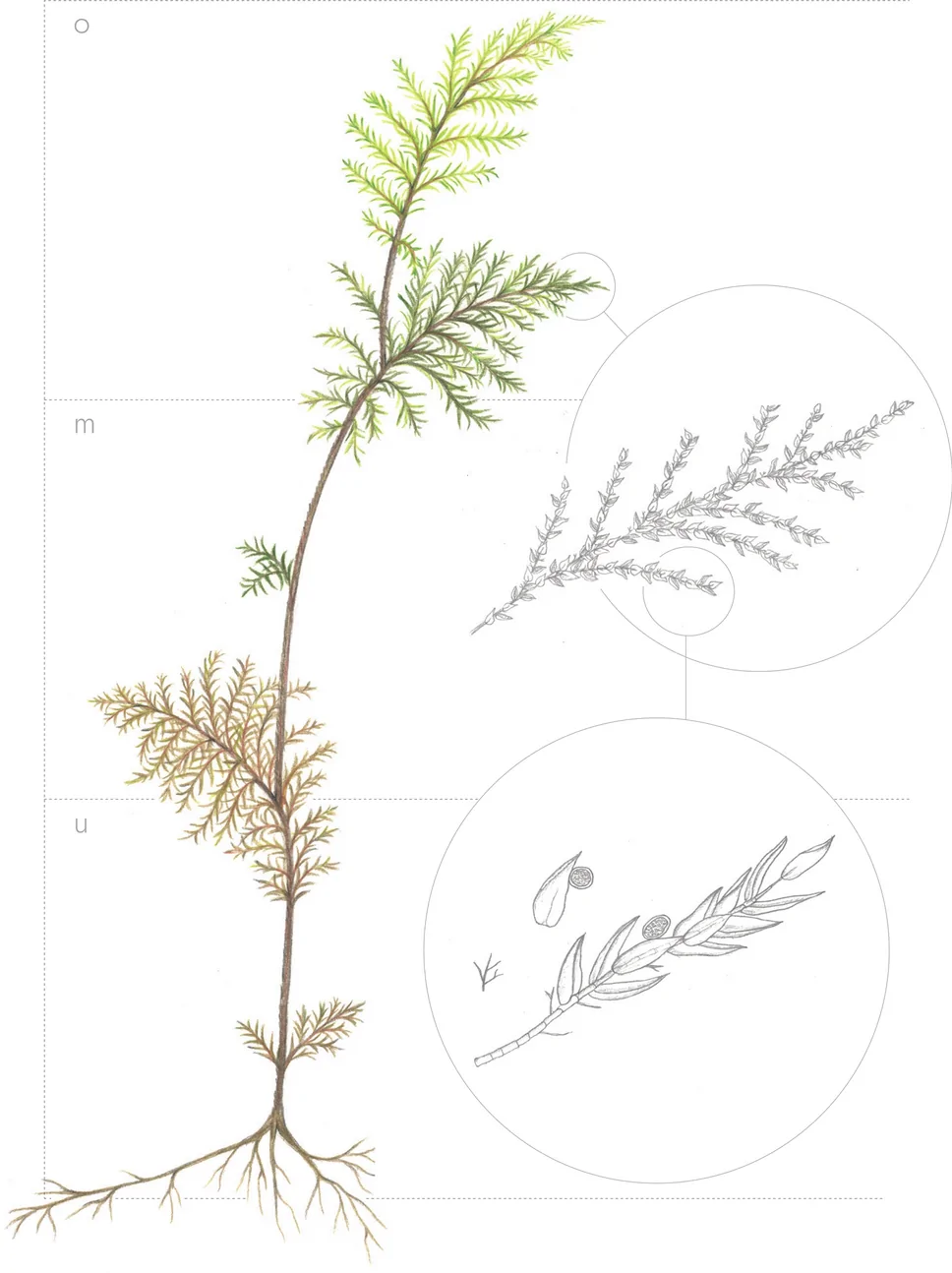
Hylocomium splendens moss with colonies of Nostoc-like cyanobacteria on leaflets (right, lower) (© J. Motzkus 2024). Plants were sectioned into three zones, from the top (labeled “oben”/“o”), middle (m) and bottom (“unten”/“u”) of caulids (Fig. 1), for comparison of relative abundances of nifH abundances among different positions

Traditionally, *Nostoc*-moss symbioses have been studied most intensively in Scandinavian and North American regions (Alvarenga and Rousk 2022), but they have been observed in many extraordinary sites throughout the world, including retreating glacial fields of Tierra del Fuego (Chile) (Arróniz-Crespo et al. 2014), Andean tropical montane cloud forest (Peru) (Permin et al. 2022) and Mount Fuji (Japan) (Kubota et al. 2023). Arboreal mosses are increasingly of interest, and have been observed to be hotspots for moss-cyanobacterial symbioses (Lindo and Whiteley 2011). Despite its proximity to Scandinavia and despite many shared moss species and sites of similar environmental conditions, direct observations of the symbiosis of feather mosses and *Nostoc*-like organisms are rare from the Central European region. To our knowledge the nostacacean symbiosis has been observed in German mosses only once, as one member out of many nitrogen-fixing bacteria associated with log-dwelling moss species, using *nifH* metabarcoding (Groß et al. 2024). Traditional “model” species of feather moss involved in nostocacean symbioses like *Hylocomium* (Fig. 1) or *Pleurozium* have to date received very little attention in central Europe, despite the prevalence of these moss species in montane forests of central Europe. Particularly likely to host moss-nostocacean symbioses are spruce- or pine-dominated forests, as *Pleurozium schreberi* or *Hylocomium splendens* are among the dominant floor mosses in these forests (Ellenberg 1988).

Molecular tests for the presence of cyanobacteria in mosses typically yield positive results and often indicate high metabolic activity, including in non-*Sphagnum* mosses (Lindo and Whiteley 2011; Warshan et al. 2016; Groß et al. 2024). Direct microscopic observation and cultivation of the symbionts are relatively rare compared to the abundance predicted by molecular data, however. Microscopic observation and cultivation of plant-symbiotic organisms can be difficult or uncertain, requiring many hours of effort and time, possibly discouraging researchers. Compounding the difficulties, *Nostoc*-like organisms and other cyanobacteria may also exist on mosses in few-to-single cell states such as hormogonia (Büdel et al. 2024) which are probably mostly missed by visual inspection, and which are difficult to render into axenic culture. We hypothesize that another factor may also contribute to the imbalance between visual observations of cyanobacterial symbionts in moss and reports of relatively high abundance and activity: some cyanobacteria may be capable of living endophytically in mosses. This is supported by numerous studies that report the presence of putative endophytic bacteria in non-*Sphagnum* mosses (e.g. – Tveit et al. (2020)). As moss leaflet lamina are only one cell thick in cross-section, we hypothesize that if such endophytic colonization occurs it is within the caulids, which are multicellular in cross-section.

Here we sought to confirm the presence of the moss-nostocacean symbioses in the region of Franconia, Germany, and to further explore the known host range for the symbiosis. Special attention was given to arboreal epiphytic mosses, especially in the family Orthotrichaceae despite never having been documented as hosts for the symbiosis. Members of Orthotrichaceae are best known as epiphytic mosses from tree bark (Fialová et al. 2023), in our region especially on broad-leaved trees with many species. We hypothesized them as likely candidates to host the symbiosis, as we hypothesize that biologically available nitrogen abundances are highly dynamic in tree canopies. Our reasoning is as follows: atmospheric nitrogen deposition in central Europe is quite high (Lüers et al. 2017), but despite this biological nitrogen fixation by moss microbiomes has been observed (Groß et al. 2024). We hypothesized therefore that abundance of biologically available nitrogen in the micro-habitat of forest mosses — and especially epiphytic mosses — is highly dynamic, following a boom-or-bust pattern. At times nitrogen is probably abundant, such as during rain events when throughfall and stemflow leaches ammonium salts and DON downward and through moss tissues (Salemaa et al. 2020). At other, drier periods when the main source of N is limited to atmospheric deposition, these relatively small mosses are small “targets” to receive such stochastic deposition, and thus may benefit from the presence of N-fixing microbes.

We hypothesized that cyanobacteria are active in moss tissues even when colonies of cyanobacteria are not visible on the moss, possibly due to endophytic colonization by cyanobacteria. We tested this by PCR-amplification of cyanobacterial-specific *nifH* genes on moss tissues with no visual colonization by cyanobacteria, and via surveys with fluorescence microscopy for cyanobacterial phycobilisomes on mosses that had been subjected to a rigorous reduction of epiphytic flora. In the case of mosses which did show obvious colonization by *Nostoc-*like organisms, we utilized a microscopy- and culture-based approach to confirm their presence, which we hoped would also enable precise imaging and taxonomic placement of symbionts, using full-length 16S and partial *nifH* PCR amplicon sequence data. We ask the following questions:

- Are nitrogen fixing cyanobacteria growing in symbioses with feather mosses such as *Hylocomium splendens* in the mountain ranges of central Europe, just as they are commonly observed to do in boreal forests?
- Are there nitrogen fixing cyanobacteria on mosses from other groups beyond feather mosses, such as the families Orthotrichaceae (*Orthotrichum* and *Zygodon* spp.) and Neckeraceae (*Homalia trichomanes*)?
- Are moss-cyanobacterial symbioses present on tree-epiphytic mosses in central European forests?
- Which species of *Nostoc* and *Nostoc-*like organisms are present in these mosses, and what are their evolutionary relationships within Nostocaceae?
- Given the predicted abundances of these symbioses, where are symbiotic cyanobacteria located on their host mosses? Are some cyanobacteria present as endophytes?

## Materials and methods

### Field

Mosses from multiple sites through the region of upper and middle Franconia (Germany) were opportunistically sampled for *Nostoc*-like organisms, as part of ongoing moss diversity inventories in the region. Mosses were taxonomically identified according to Frey et al. (2021). Moss specimens were collected when visual inspection with a 10× lupe indicated possible cyanobacterial colonies, in the form of blue discoloration. Moss samples were collected from multiple different locations in the Fichtel Mountains, the Franconian Jura, and other sites at elevations between 350 and 800 m above sea level. Sample collections were carried out as part of other ecological surveys which took place during the years 2023—2025, see Table 1 for details. Each moss sample consisted of at least one healthy gametophyte individual (all continuous tissue from the rhizoids to the shoot). Samples were stored in plastic bags and kept in the dark at 4 °C before further use.

**Table 1:** Sampled locations and moss species

Locality name	Latitude/longitude (°)	Moss species	substrate	Elev (m)	Sample date
Fichtelgebirge, Waldstein	50,14,233 11,86,628	*Hylocomium splendens*	Soil	774	May-2023 May-2024
Ecological Botanical Garden, University of Bayreuth	49,92,438 11,58,454	*H. splendens*	Soil	354	May-2023
Northern Fraconian Jura, near Neustädtlein	49,96,020 11,43,589	*Orthotrichum lyellii*	Bark (*Fraxinus excelsior)*	480	Nov-2024
Southern Fraconian Jura, Pfaffenberg near Mettendorf	49,02075 11,35,922	*Zygodon dentatus*	Bark (*Sorbus schuwerkiorum*)	470	Nov-2024
Northern Fraconian Jura, Schwarzachklamm near Gsteinach	49,352,118 11,232,484	*Homalia trichomanoides*	Bark (*Fagus sylvatica*)	345	May-2025
Southern Fraconian Jura, Pfaffenberg near Mettendorf	49,02075 11,35,922	*Pseudoambly-stegium subtile*	Bark (*Sorbus schuwerkiorum*)	470	Nov-2024

In Orthotrichum, Nostoc-like organisms were photographed but failed to culture. Samples from University of Bayreuth were used for PCR tests for nifH but not for microscopy or culturing. All other mosses were subject to microscopic inspection for- and culturing of Nostoc-like organisms

Graphical overviews of laboratory and bioinformatics are available (supp. fig. 1 and 2).

### Microscopy

Preliminary surveys for the presence of *Nostoc* were conducted with standard light microscopy, first using binocular stereoscope (MDG-17, Wild HeerBrugg, Switzerland), followed by compound light microscopy (Leitz HM-Lux 3 and Revolve 2, ECHO, San Diego, USA). For comparison, some specimens which were surveyed for *Nostoc-*like colonies using light microscopy and which were found not to host visible colonies were then subjected to survey for endophytic cyanobacteria (whole-community DNA extraction and PCR, see next). Sectioning was done using sections of one centimeter from the top (o), middle (m) and bottom (u) of caulids (Fig. 1), for comparison of relative abundances of *nifH* abundances among different positions. Sectioning was done with a scalpel that was flame-sterilized over a Bunsen burner, before and after cuts. Samples with *Nostoc-*like colonies were then subjected to further investigation with epifluorescence microscopy.

Moss tissues and bacterial cultures were observed under an epifluorescence microscope (Revolve 2, ECHO, San Diego, USA), using Revolve Software 4.1.0, app version v7.1.0. With epifluorescence microscopy, cyanobacteria can be easily more discerned from microscopic chlorophytes and streptophytes (Grigoryeva 2020; Wojtasiewicz and Stoń-Egiert 2016). “Cy5” filter setup (CHROMA 49009, ex: 610–650 nm, em: 660–735 nm), can be used to excite C-phycocyanins (Poniedziałek et al. 2017). Phycoerythrins can be excited using “TXRED” filter settings (CHROMA 49008, ex: 540–580, em: 590–665 nm). Algal and plant cells were included in image overlays, using the “FITC/Cy2” filter set (CHROMA 49002, ex: 450–490 nm, em: 500–550 nm) (Donaldson 2020; Tobimatsu et al. 2013).

### Epiphyte reduction

To test the hypothesis that cyanobacteria inhabit endophytic compartments of the host mosses, epiphytic flora of some *Hylocomium* moss samples were reduced using chemical means following Alvarenga et al. (2024) with some modifications. 2–3 g (about 5–10 gametophytes) of moss were manually cleaned of debris, placed into plastic 50 mL centrifuge tubes, and submerged in 40 mL of 0.5% Tween20 solution. Samples were ultrasonicated for a minimum of 2 min with a VWR ultrasonic cleaner at power 5. Liquid was removed by decanting, and replaced with 40 mL sterile de-ionized, distilled H_2_O, vortexed for 10 s, and discarded by decanting. Samples were then subjected twice to submersion in 1% NaOCl solution, and agitation by vortex mixer for 10 s at high speed. After each NaOCl application, samples were rinsed and agitated in 40 mL sterile de-ionized, distilled H_2_O. Epiphyte-reduced samples were then again inspected for visible cyanobacterial colonization.

### Culture of *Nostoc*-like organisms

The abundant chloroplast DNA present in host moss tissue complicates molecular laboratory and bioinformatic pipelines for characterizing cyanobacteria (Fitzpatrick et al. 2018; Song and Xie 2020), so we attempted a cultivation-based pipeline for describing our observed *Nostoc*-like organisms during microscopic inspection. “*Nostoc*-like organisms” were defined as any filamentous, chlorophyllous bacteria that were observed in spherical, sheathed colonies, especially when trichomes displayed obvious intercalary heterocytes. Media for all cultivation was nitrogen-free BG-11_0_ media (Rippka et al. 1979). Entire stems (0.5–2 cm length) of host moss were submerged into liquid media for 2–8 weeks in 15 mL Falcon centrifuge tubes at room temperatures with natural (window) lighting, followed by removal of moss tissue upon observation of phototrophic growth, to reduce further contamination. Artificial augmentation of both temperature and light levels were attempted but either did not produce additional growth or resulted in colony death. Most phototrophic growth observed in these samples were due to microscopic chlorophytes and streptophytes, but in some cases growth of *Nostoc*-like colonies was observed. These colonies of *Nostoc*-like organisms were then further separated into separate liquid media aliquots through trapping with pipette tips, retaining as little host tissue and microscopic chlorophytes and streptophytes as possible in the process. After 1–2 months, these colonies reproduced sufficient further colonies, which were then individually captured using micropipettes set to take up 2 µl volumes. Colonies were then transferred to new 1.5 mL centrifuge tubes, where they were subjected to a sterile water wash and subsequent submersion in fresh BG-11_0_ media in 15 mL centrifuge tubes. These isolations were allowed to grow an additional 1–2 months, then were subject to microscopy as detailed above, and colony PCR as detailed below. *Nostoc*-like organisms were successfully isolated and cultivated from 4 separate moss host species, for further DNA extraction and PCR.

### DNA extraction

Whole-community (host and microbial) DNA was extracted using the Quick-Start Protocol DNeasy®Plant Pro Kit from QIAGEN®, following manufacturer instructions. In step 1, twice the amount of CD1 was initially added, as the specified amount of added liquid was often not sufficient to rehydrate moss tissue.

For use as additional PCR controls for whole-community (moss + microbiome) *nifH*, DNA was also extracted from (1) liquid culture of *Trichormus variabilis* (DSM 107003, syn. *Anabaena variabilis*), (2) leaf and stems of epiphyte-rduced moss *Brachythecium* sp. collected from a roadside urban grass-dominated landscape in Bayreuth, Germany, that was presumed to be abundant with nitrates and therefore low in cyanobacterial nitrogen fixation activity, and (3) epiphyte-reduced leaf tissue from *Solidago* sp., a common vascular plant in the region. In both *Brachythecium* and *Solidago* control samples, epiphyte reduction was conducted in the following way: thorough rinse with sterile water, followed by immersion for 1 min in 97% ethanol soak and 30 s in 2% NaOCl, followed by additional immersion for 1 min in 97% ethanol and sterile water rinse. After extraction, the DNA was checked for quality and quantity using the NanoDrop One (Thermo Scientific) and Qubit 4 assay (Thermo Fisher Scientific) and frozen at − 20 °C to preserve it for later testing.

### PCR

To test for the presence of cryptic colonization by nitrogen-fixing cyanobacteria in moss tissues, culture-free, direct PCR-amplification of cyanobacterial *nifH* genes from DNA of whole-community mosses (moss+microbiome) was conducted on samples of *H. splendens* collected from the University of Bayreuth Ecological Botanical Garden (see Table 1). These moss samples had been inspected via standard light microscopy and had shown no evidence of cyanobacterial colonization. The CNF (5 ‘-CGTAGGTTGCGACCCTAAGGCTGA-3 ‘) and CNR (5 ‘-GCATACATCGCCATCATTTCACC-3 ‘) primers (Olson et al. 1998) were used as per Díez et al. (2007). These primers are expected to amplify sequences of 359 bp length when used on cyanobacterial DNA. Reactions were conducted in 25 μL volumes: 12.5 μL NEB Q5® High-Fidelity 2X Master Mix, 0.3 μL BSA (20 ng/μL), 1.2 μL forward and reverse primers each (10 μM), 7.3 μL PCR-grade H_2_O and 2.5 μL of template DNA. PCR conditions were as follows: initial denaturation at 98ºC for 2 min, followed by 32 cycles of 98ºC denaturing for 15 s, 50ºC annealing for 30 s and 72ºC extension for 30 s, and a final extension of 72ºC for 7 min. *nifH* PCR amplicons from whole-community moss samples were subject to gel visualization but were not further sequenced, due to an apparent abundance of *nifH* homologs (Angel et al. 2018) from host mosses (see results). *Hylocomium* samples from University of Bayreuth were not used in any further analysis in this study.

Isolation and growth of *Nostoc*-like colonies was extremely slow and yielded insufficient biomass for DNA extraction. Thus, for identification and taxonomic placement of successfully cultured *Nostoc-*like organisms, direct colony PCR of individual *Nostoc* colonies was necessary to target and amplify full length 16S rRNA and *nifH* genes. Individual *Nostoc*-like spherical colonies were trapped via suction onto 20 μL pipette tips, washed in sterile water, and placed directly into 50 μL reaction volumes.

PCR-amplification of *nifH* genes from cultures used CNF/CNR primers (see above). PCR reagents were mixed as follows, per 50 μL reaction: 10 μL NEB Q5® 5× reaction buffer, 10 μL NEB Q5® High GC enhancer, 0.5 μL NEB Q5® HiFi polymerase, 1.0 μL mixed dNTPs (10 mM), 0.6 μL BSA (20 ng/ μL), 1.2 μL forward and reverse primers each (10 μM), 4 μL of template DNA, and PCR-grade H_2_O to 50 μL total volume. PCR conditions for amplification of *nifH* were as follows: initial denaturation at 98ºC for 5 min, followed by 30 cycles of 98ºC denaturing for 15 s, 50ºC annealing for 30 s and 72ºC extension for 30 s, and a final extension of 72ºC for 7 min.

For PCR-amplification of 16S rRNA genes from cultures, protocol followed Cuscó et al. (2019), with modifications for the colony-PCR. Primers 16S-27F (5’-AGRGTTTGATYHTGGCTCAG-3’) (Zeng et al. 2013) and 16S-1492R (5’-TACCTTGTTAYGACTT-3’) (Klindworth et al. 2013) were used, targeting the majority of the 16 s rRNA gene. PCR reagents were mixed as follows, per 50 μL reaction: 10 μL NEB Q5® 5× reaction buffer, 10 μL NEB Q5® High GC enhancer, 0.5 μL NEB Q5® HiFi polymerase, 1.0 μL mixed dNTPs (10 mM), 0.4 μL BSA (20 ng/μL), 2.5 μL forward and reverse primers each (10 μM), 4 μL of template DNA, and PCR-grade H_2_O to 50 μL total volume. PCR conditions were as follows: denaturation at 98ºC for 4 min amplification, followed by 30 cycles of 98ºC for 15 s, 51ºC annealing for 30 s, and 72ºC extension for 1 min, and a final extension of 72ºC for 7 min.

PCR products from both *nifH* and 16S rRNA amplifications were cleaned using a Nucleospin Gel and PCR cleanup column kit (Macherey–Nagel, www.mn-net.com, article-number: 740609.50), with eluted to a 14 μL volume, with final DNA concentrations between 3.8 ng/μL—40.2 ng/μL.

### Sequencing of *nifH* and 16S rRNA genes

Colony-PCR products from *Nostoc*-like cultures were then prepped according to manufacturer instructions for Nanopore-platform sequencing using either (1) the Oxford Nanopore Technologies (ONT) ligation sequencing kit V14 (SQK-LSK114) or (2) ONT Rapid sequencing V14 -Amplicon sequencing (SQK-RBK114.24), with a R10.4.1 (FLO-MIN114) generation flow cell. SQK-LSK114 kit was used in the case of 16S rRNA gene of *Hylocomium*-associated *Nostoc* from Waldstein, which was sequenced first and separately from later cultures. PCR of *nifH* gene from *Pseudoamblystegium*-associated *Nostoc*-like organism was not successful. The remaining 6 colony-PCR products to be sequenced consisted of *nifH* and 16S rRNA amplicons from *Nostoc-*like organisms successfully cultured from *Orthotrichum*, *Zygodon*, and *Homalia* mosses, and the *nifH* amplicons from the *Hylocomium-*associated *Nostoc-*like organism from Waldstein. These were individually barcoded, then multiplexed and sequenced jointly using a SQK-RBK114.24 kit. In the first sequencing run, which included only 16 s rRNA amplicons from the *Hylocomium*-associated *Nostoc-*like organism, sequencing ran until a depth of ~100,000 reads, with simultaneous fast-model basecalling using Guppy (MinKNOW v23.07.12, Bream v7.7.6, Guppy v7.1.4, MinKNOW Core v5.7.5). In the second, multiplexed sequencing run sequencing was run until total depth of ~38,000 reads, without basecalling, which was conducted separately with Dorado basecalling software (MinKNOW v25.05.12, Bream v8.5.4, Dorado v7.9.8, MinKNOW Core v6.5.13). In both cases default minimum Q-scores of 8 were used to define passing reads.

## Bioinformatics

### Processing raw reads to consensus sequences

Demultiplexing and trimming of adapters and barcodes of Nanopore reads from colony-PCRs of *Nostoc*-like cultures was done using the Dorado basecalling softare. Resulting reads were then re-oriented 5’-3’ where necessary, using VSEARCH (Rognes et al. 2016) v2.30.0, against the Silva 138.2 (Quast et al. 2013) Genus-level training dataset, formatted for DADA2 software (“silva_nr99_v138.2_toGenus_trainset.fa”, available at www.arb-silva.de/current-release/DADA2/1.36.0/SSU). Re-orientation of *nifH* reads was conducted against the custom *nifH* database of *nifH* sequences from Kubota et al. (2023). In the case of the sequence library that was basecalled with Dorado, the Dorado command executes includes a default chimera-check/read-splitting step for chimeric reads resulting from Nanopore sequencing error. In either case, PCR-chimeras were screened manually in the alignments discussed below.

Resulting reads from nanopore sequencing were typically numerous and very noisy, aligning poorly due to numerous erroneous insertions, paralogs, and co-cultured microorganisms. In some cases, BLAST (Basic Local Alignment Search Tool)-family algorithms (Altschul et al. 1990) have been found to perform effectively for aligning reads with common error patterns of long-read metabarcoding data (Tedersoo et al. 2021, 2022). Therefore, as a first filtering step before full alignments, we used the BLAST+ suite tools, version 2.9.0 (Comacho et al., 2009), specifically blastn to retain only high-quality matches to nostocacean 16S sequences from the Silva 138.2 database (see link above), and blastx to retain high-quality matches of our *nifH* DNA sequences to sequences from a custom database of NifH amino acid sequences, to remove possible *nifH* homologs. Following Hu et al. (2024), our custom database of NifH sequences was a concatenation of NifH sequences from (1) 963 culture-derived genomes (Koirala and Brözel 2021), the Greening lab NifH database (https://doi.org/10.26180/c.5230745) and the NIFtHool database (Suquilanda-Pesántez et al. 2022). Following this filtering step, reads were aligned de novo using MUSCLE 5.1 with default settings (Edgar 2022). Vertical gaps were removed using CIAlign v1.1.4 (Tumescheit et al. 2022), using a insertion minimum percentage of 70%, of all gap sizes (insertion minimum size > 0, insertion minimum flank size > 0). At this stage, further screening for chimeric reads was conducted: over-length reads were discarded, and remaining possible chimeras were discovered via hand-curation of the alignments.

Both 16S (Sun et al. 2013) and *nifH* (Thiel 1993; Thiel et al. 1995) can be present in multiple unique copies within a single organism. To detect this possible paralogous, intra-genomic variation, trees of full alignments of amplicon sequences were constructed using FastTree 2.2 with default settings (Price et al. 2010), and visualized with ARB 7.0 (Ludwig et al. 2004). Three reference sequences of *nifH* were extracted from the genome of *Nostoc punctiforme* PCC 73102 / ATCC 29133 (NCBI Reference Sequence NC_010628.1) and included in this alignment. These reference *nifH* genes are annotated/positioned in the genome as follows: WP_012407220.1 (position 512,909–513802), WP_012407784.1 (position 1,229,540–1230433), WP_012412139.1 (position 7,297,077–7298042). Unrooted trees were searched for obviously separate clades (example shown in Supp. Fig. 3). If present, these reads from these subtrees were again aligned individually using MUSCLE, and manually curated for removal of poorly aligned sequences and ends. If obviously separate clades were not present in the phylogenetic tree, the full alignment of 16 s or *nifH* was assumed to represent a single representative gene sequence for that organism. In either case, consensus sequences were generated using Hidden Markov models (HMM) from each (sub)alignment, using the hmmbuild and hmmemit commands, from the HMMER v3.3 package (Eddy 2011), using simple majority-rule decision for consensus-sequence bp calls. Since *nifH* Nanopore sequences were not preliminarily taxonomically filtered to *Nostoc*, unlike 16S (see above), resulting *nifH* consensus nucleotide sequences were at this point searched against the full NCBI Core nucleotide BLAST database (blast.ncbi.nlm.nih.gov), for highly similar matches using the online blastn graphical user interface, to confirm likely Nostocaceae origin.

### *nifH* phylogeny

Our *nifH* phylogenetic analysis was intended to follow—and build upon—Ininbergs et al. (2011) and Kubota et al. (2023). All pertinent *nifH* sequences from both studies were provided by authors (Masayuki Kubota, pers. comm.), combined with *nifH* consensus sequences from our cultures. Alignment was conducted using Muscle v5.1 with default settings, and tree was constructed using FastTree2 using default settings.

### 16S Nostocaceae phylogeny

For taxonomic identification of full-length 16S consensus sequences from cultured *Nostoc*-like organisms, a de novo phylenogenetic tree of full-length 16S sequences for Nostocaceae was constructed. The full Cyanoseq (Lefler et al. 2023) v1.2 phylogenetic tree (https://zenodo.org/records/7864137) was visualized in ARB v7.0, and sequences of all members of *Nostoc*aceae were selected, including sequences from closely allied clades Tolypotrichaceae, Aphanizomenaceae, and Nodulariaceae (Strunecký et al. 2023). Additionally, 708 sequences of full or near-full length 16S from NCBI genbank that were identified as high-quality *Nostoc* sequences by Cyanoseq v1.3 were provided by the authors for use in our 16S tree (Forrest Lefler, pers. comm; full cyanobacterial dataset available at https://zenodo.org/records/13910424), along with several of 16S reference sequences of interest: *Trichormus azollae* (NC_014248.1, syn. *Nostoc azollae*) (Ran et al. 2010), *Nostoc calcicola* SAG 1453–1 (KM019926) and *Nostoc insulare* SAG 54.79 (KM019927). Full 16S sequence of *T. azolla*e was not available as a stand-alone NCBI nucleotide accession, so 16S copies were extracted from reference genome with barrnap (https://github.com/tseemann/barrnap). *Scytonema pachmarhiense* (MH260366) was added as an outgroup for rooting of phylogenetic trees. In total, 856 sequences were included in our *Nostoc*aceae tree. Reads were aligned using SSU-align v0.1.1 (Nawrocki 2009), using default Bacterial models. Tree was constructed using FastTree2. Full alignment FASTA and tree Newick files are available in supplementary materials. Tree topology was visualized with ARB 7.0, and final cosmetic additions to tree labels and highlighting of taxa of interest were done using Inkscape v1.3.2 for GNU linux.

## Results

### Microscopy

**Fig. 2:**
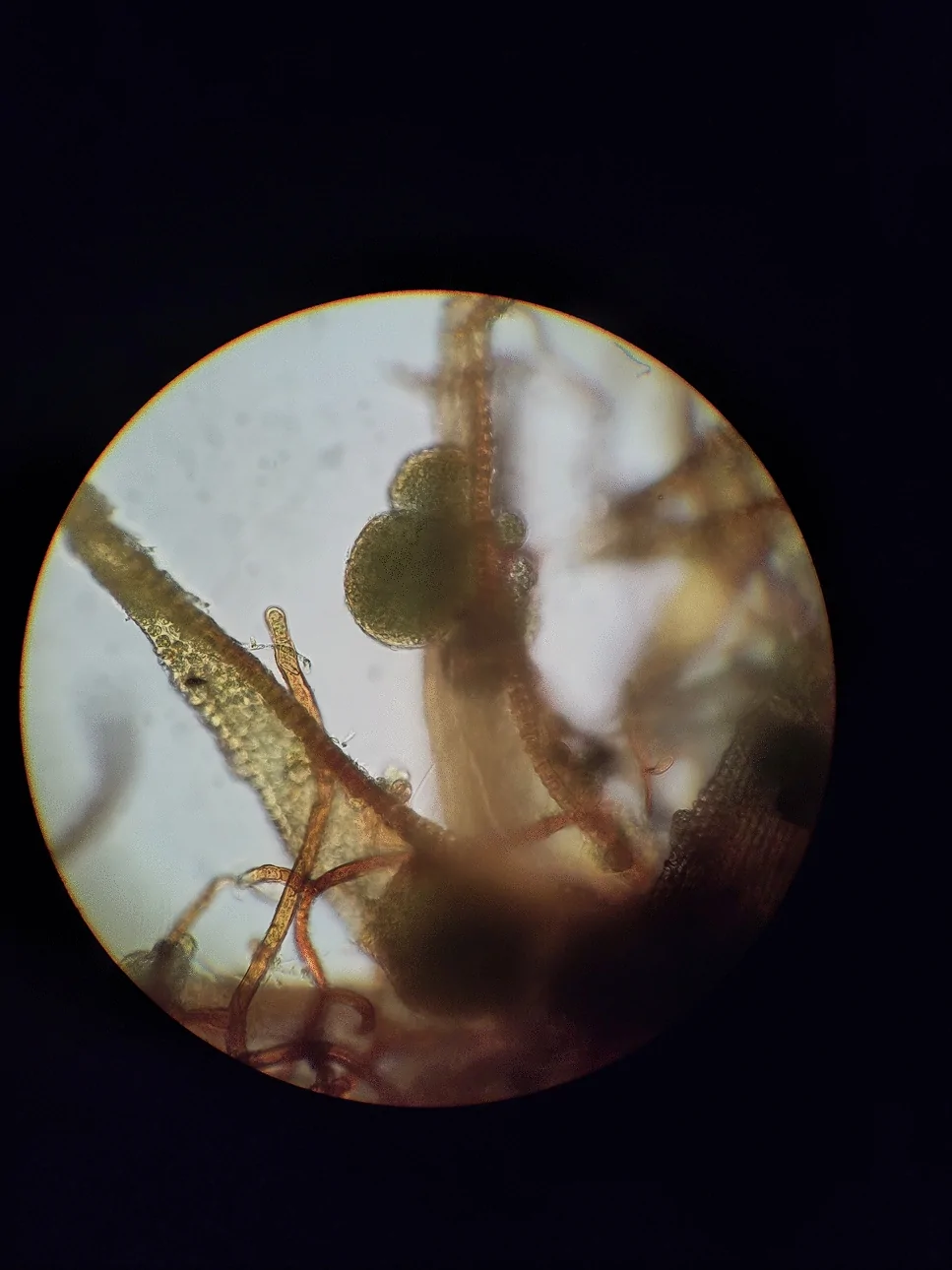
Colonies of *Nostoc*-like organism observed from *Orthotrichum lyelli* at 400× magnification

**Fig. 3:**
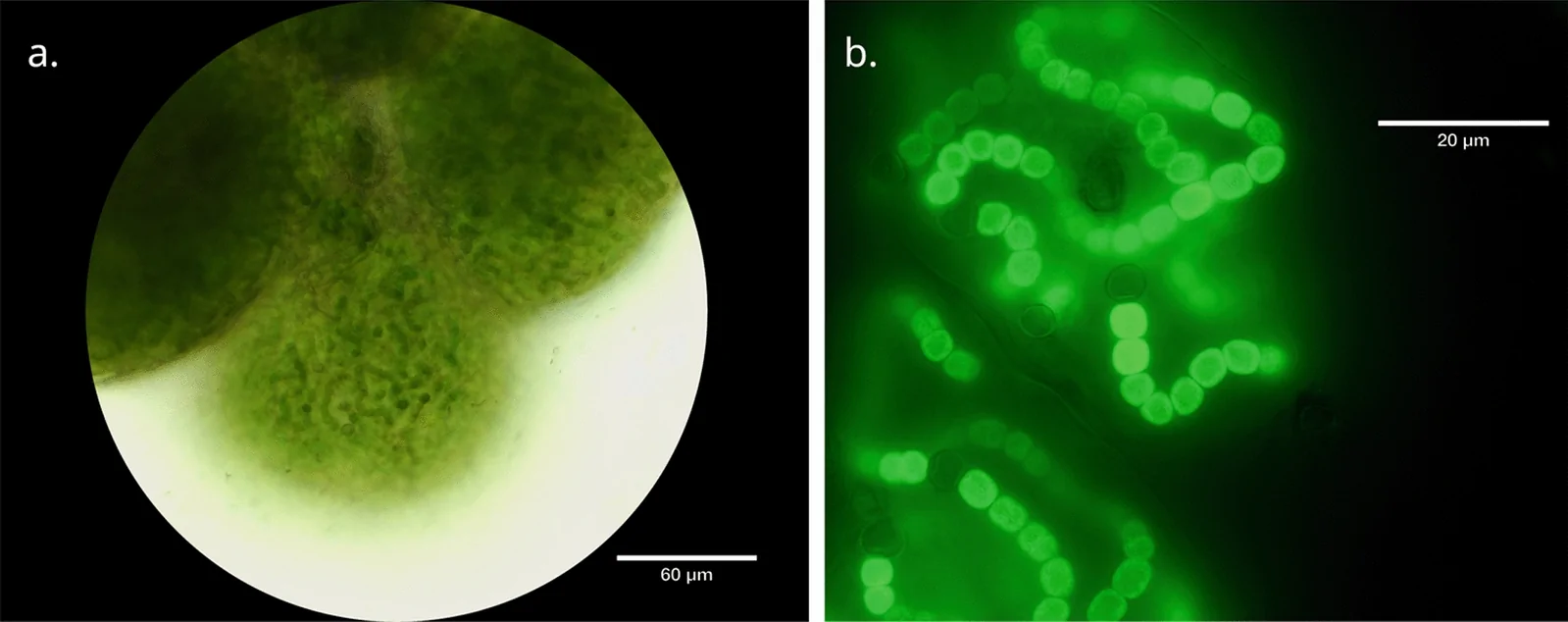
Colonies of *Nostoc*-like organism after isolation from *Hylocomium splendens*. (**a**) light microscopy, at 400× magnification and (**b**) 1000× magnification using epifluorescence with Cy5 filter setup (see methods), showing trichomes and heterocysts

**Fig. 4:**
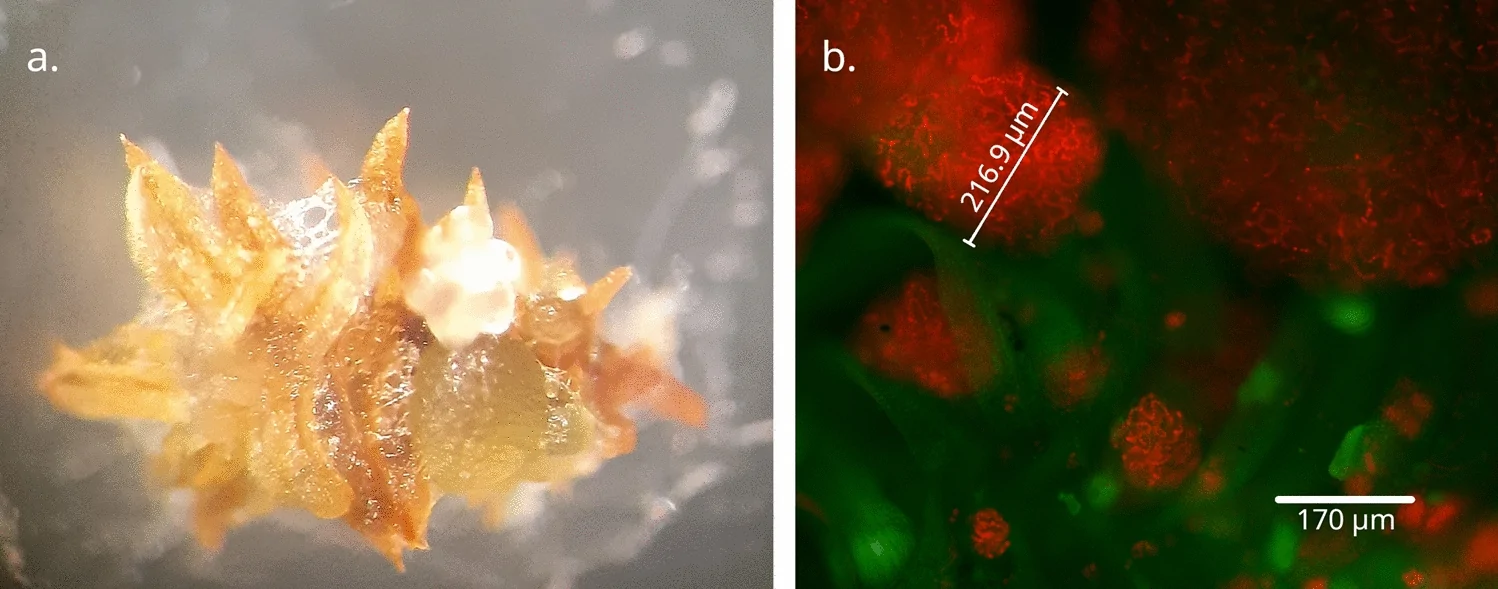
Colonies of *Nostoc*-like organism observed from *Zygodon dentatus* (**a**) at 50× magnification with binocular dissecting microscope show heavy colonization of moss caulid. (**b**) at 400×, same colonies using epifluorescence with Cy5 filter setup for detection of cyanobacteria, overlaid onto FITC setup for background moss tissue (see methods)

**Fig. 5:**
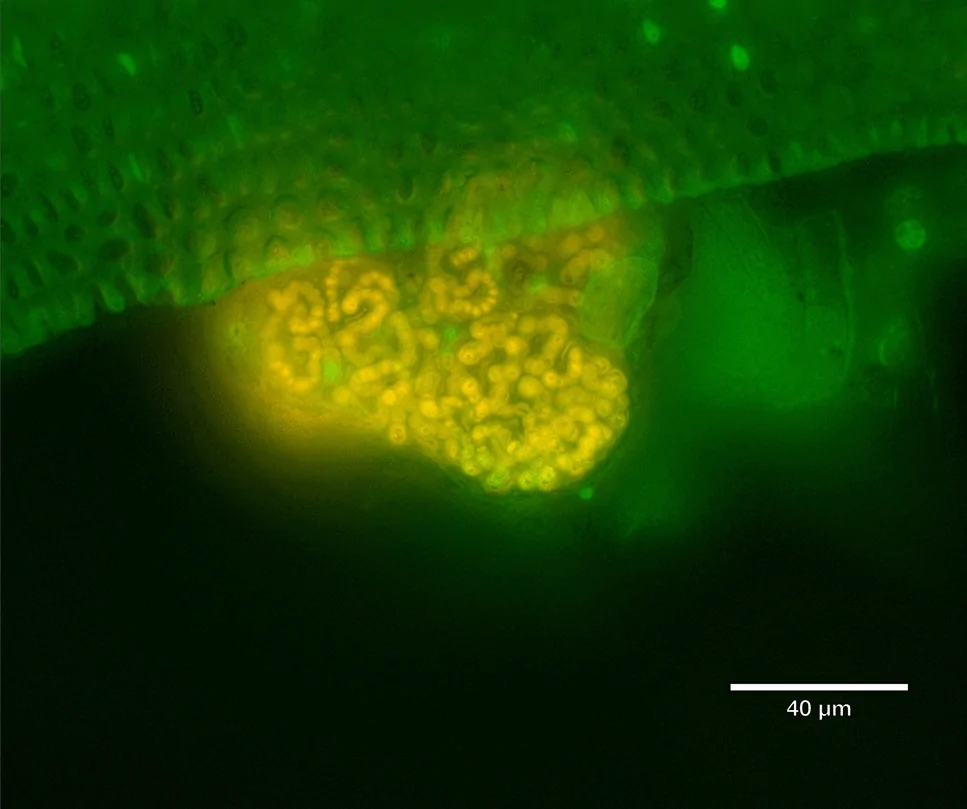
Colonies of *Nostoc*-like organism observed from *Homalia trichomanoides* at 400× using epifluorescence with TXRED filter setup for detection of cyanobacteria, overlaid onto FITC setup for background moss tissue (see methods)

**Fig. 6:**
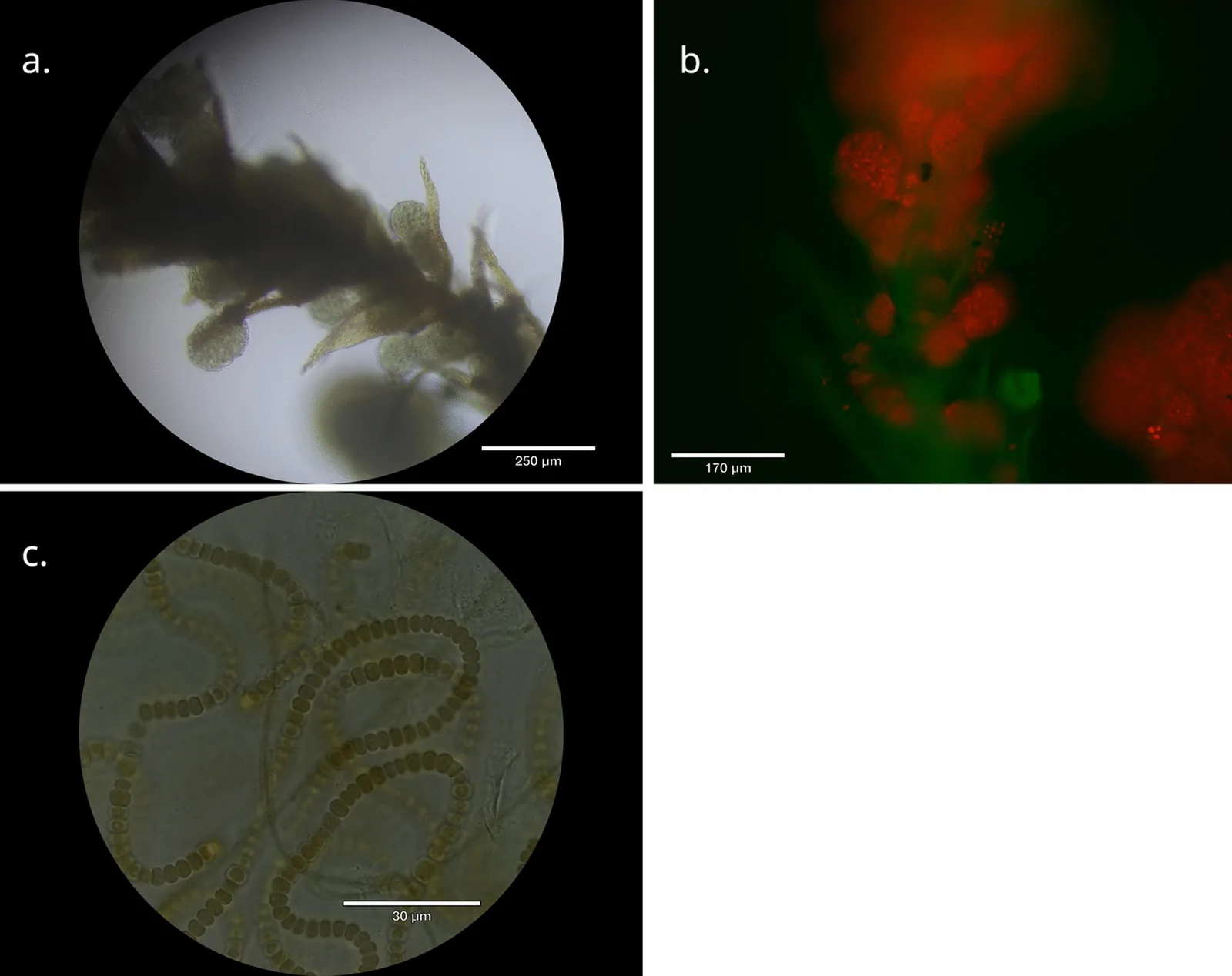
Colonies of *Nostoc*-like organism observed from *Pseudoamblystegium subtile*. (**a,b**) abundant colonization by *Nostoc*-like colones on caulid at 100× magnification with light and using epifluorescence with Cy5 filter setup for detection of cyanobacteria, overlaid onto FITC setup for background moss tissue (see methods). (**c**) 1000× magnification of same organism, showing trichomes and heterocysts

*Nostoc*-like organisms were observed in 5 moss species, from 4 sites (Figs. 2, 3, 4, 5 and 6):

*Orthotrichum lyelli* from Northern Fraconian Jura near Neustädtlein*, Hylocomium splendens* from Waldstein*, Zygodon dentatus* and *Pseudoamblystegium subtile* from Pfaffenberg near Mettendorf, and *Homalia trichomanoides* from Schwarzachklamm near Gsteinach. Within each moss, position of *Nostoc-*like colonies were variable but were most common in the leaflet axils of the moss gametophytes. Most notably in *Orthotrichum lyelli,* the position of *Nostoc–*like colonies were more variable, occurring in the leaf axils but also often on leaf laminas.

### Epiphyte reduction

**Fig. 7:**
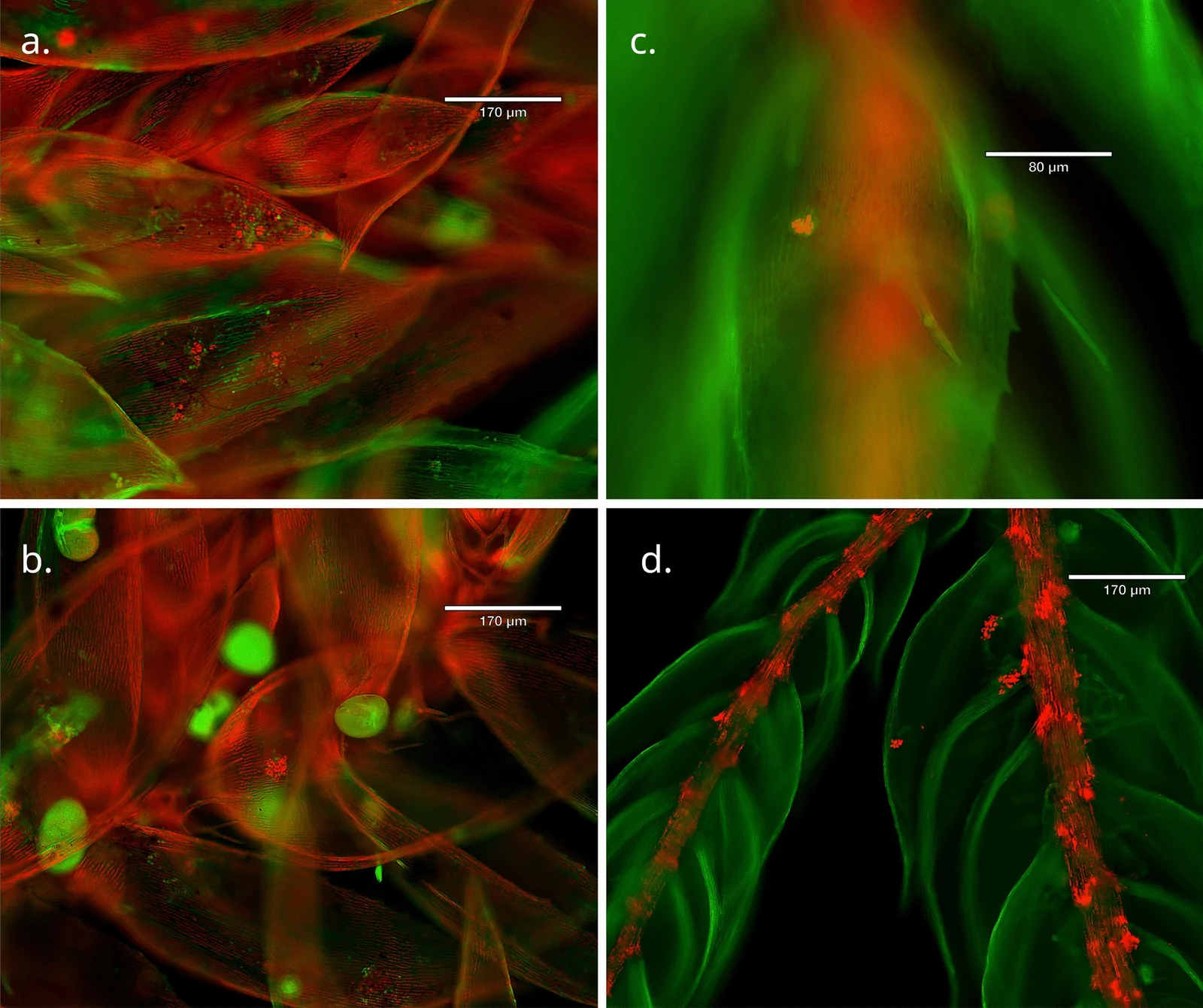
Microbiota of leaves and stems of Hylocomium splendens, using epifluorescence with Cy5 filter setup for detection of cyanobacteria, overlaid onto FITC setup for background moss tissue (see methods). (**a-b**) fluorescence of cyanobacteria and microscopic chlorophytes and streptophytes on untreated moss leaves. (**c,d**) after epiphyte reduction, microscopic chlorophytes and streptophytes and cyanobacterial cells on leaves are mostly no longer visible, but colonies on stems remain visible, possibly indicating endophytic lifestyle within stems

Epiphyte reduction of *Hylocomium splendens* fronds greatly reduced the amount of visible colonization by cyanobacteria and other phototrophs (Fig. 7). Generally, epiphyte reduced leaf surfaces were cleared of visible colonies, though occasionally some microbial cells were observed (Fig. 7c). *H. splendens* stems still appeared to consistently harbor extensive significant microbial biomass following epiphyte reduction, possibly cyanobacteria because of their fluorescence patterns (Fig. 7d). Post-treatment fluorescence signals are most concentrated at the leaflet-axils, but are also observed across the remaining area of caulids.

### Culture of *Nostoc*-like organisms

*Nostoc*-like organisms were successfully cultured from 4 out of the 5 moss gametophytes that were visually confirmed to host *Nostoc*-like organisms. Successful *Nostoc-*like isolates were from: *Hylocomium splendens* (Fig. 3)*, Homalia trichomanoides* (Fig. 5)*, Pseudoamblystegium subtile* (Fig. 6)*, and Zygodon* sp. (Fig. 4)*.* The *Nostoc-*like organism from *Orthotrichum* moss (Fig. 2) failed to show any growth and typically became chlorotic, despite abundant growth on the collected moss tissue. The *Pseudoamblystegium-*associated *Nostoc-*like organism was lost shortly after transfer into liquid culture, and was overtaken by an unidentified, co-cultured non-filamentous cyanobacterium. This may have resulted in a failed PCR amplification of *nifH*, though some 16S rRNA gene sequence data from the *Nostoc*-like organism were recovered (see below).

### Whole-community amplification of *nifH* gene

Culture-free amplification of *nifH* using cyanobacterial *nifH* primers from *Hylocomium* moss tissue consistently showed amplification from moss tissues where no cyanobacterial colonization was visually detected (supp. fig. 4). Two bands in the size range of interest in the whole-community (moss + microbiome) samples were consistently observed. One band matched the expected size of *nifH* and matched bands presumed to *nifH* amplicons observed from *T. variabilis-*only controls, and appeared in all moss samples and sections at varying brightnesses. Another slightly larger size class band was displayed which appeared only when a host plant was present and not in amplicons from axenic culture of *T. variabilis*, possibly consisting of hosts plant homologs to *nifH*. Other, much larger amplicons were also observed, likely due to non-optimized PCR conditions, probably consisting of either non-specific amplification or concatomeric PCR products.

### Phylogenetic placement of *nifH *genes from cultured *Nostoc*-like organisms

**Fig. 8:**
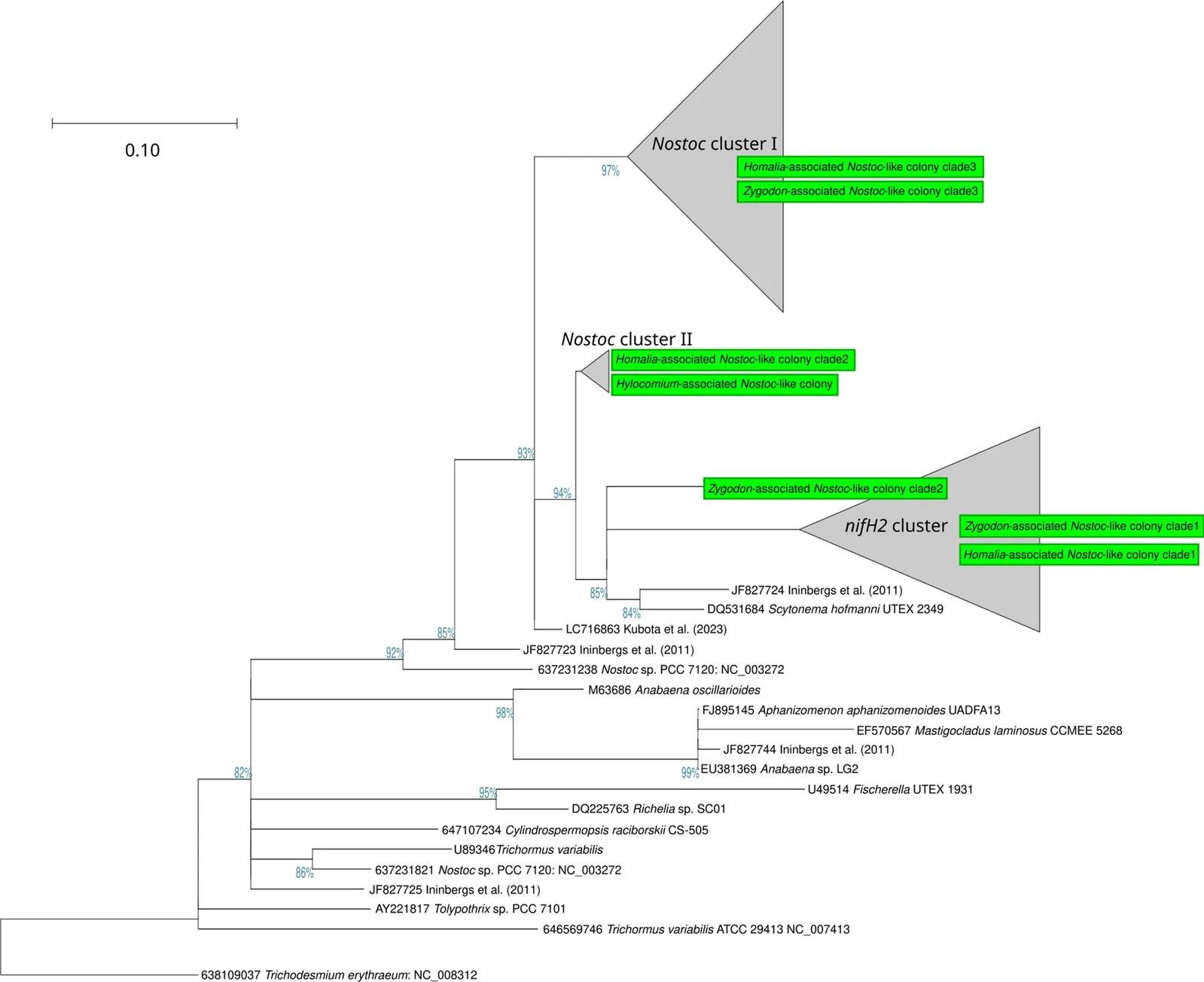
nifH gene phylogeny of select members of Nostoceae. Tree is constructed from de novo alignment of nifH sequences detected in this study from Nostoc-like organisms (green) and reference sequences from Ininbergs et al. (2011) and Kubota et al. (2023). Zygodon- and Homalia-associated Nostoc-like organisms both hosted multiple distinct copies of nifH. Branch lengths are proportionate to expected number of nucleotide substitutions per site. Branch support values are Shimodaira-Hasegawa (SH) test values, ranging from 0 (low confidence) to 1 (high-confidence). Full-size tree with all clades expanded, including placement of “Stigonema Cluster”, is available as Supp. Fig. 5

We detected *nifH* sequences from 3 of the 4 *Nostoc*-like organisms: *Hylocomium-, Zygodon-,* and *Homalia-*associated *Nostoc-*like organisms. Despite differentiated cells resembling heterocysts (Fig. 6), PCR-amplification of *nifH* from our culture of *Pseudoamblystegium-*associated *Nostoc-*like organism did not yield any sequence-able product, probably due to overgrowth by a non-nitrogen fixing cyanobacteria (see above). The topology of our *nifH* tree recaptured the major clades of Kubota et al. (2023) and Ininbergs et al. (2011) with some minor differences (Fig. 8, supp. fig. 5). The most important distinction was the inclusion of the *Stigonema nifH* cluster within *Nostoc* cluster I. *Zygodon-* and *Homalia-*associated cultures each appeared to host 3 alternative nitrogenases, several of which showed homology respectively with alternate *nifH* sequences extracted from the reference genome of *Nostoc punctiforme PCC 73102 / ATCC 29133* (supp. fig. 5). In each case, these observed alternate *nifH* versions were placed into separate clades on the Kubota-Ininbergs *nifH* tree, even though originating from a single colony, and likely from single strain of *Nostoc-*like organism. Far fewer high-quality *nifH* sequences were recovered from the culture of our *Hylocomium-*associated *Nostoc*-like organism, in comparison to our two other *Nostoc-*like cultures (supp. fig 3), but these were sufficient to generate a single candidate consensus sequence for *nifH* in this organism.

### Phylogenetic placement of 16S genes from cultured *Nostoc*-like organisms

**Fig. 9:**
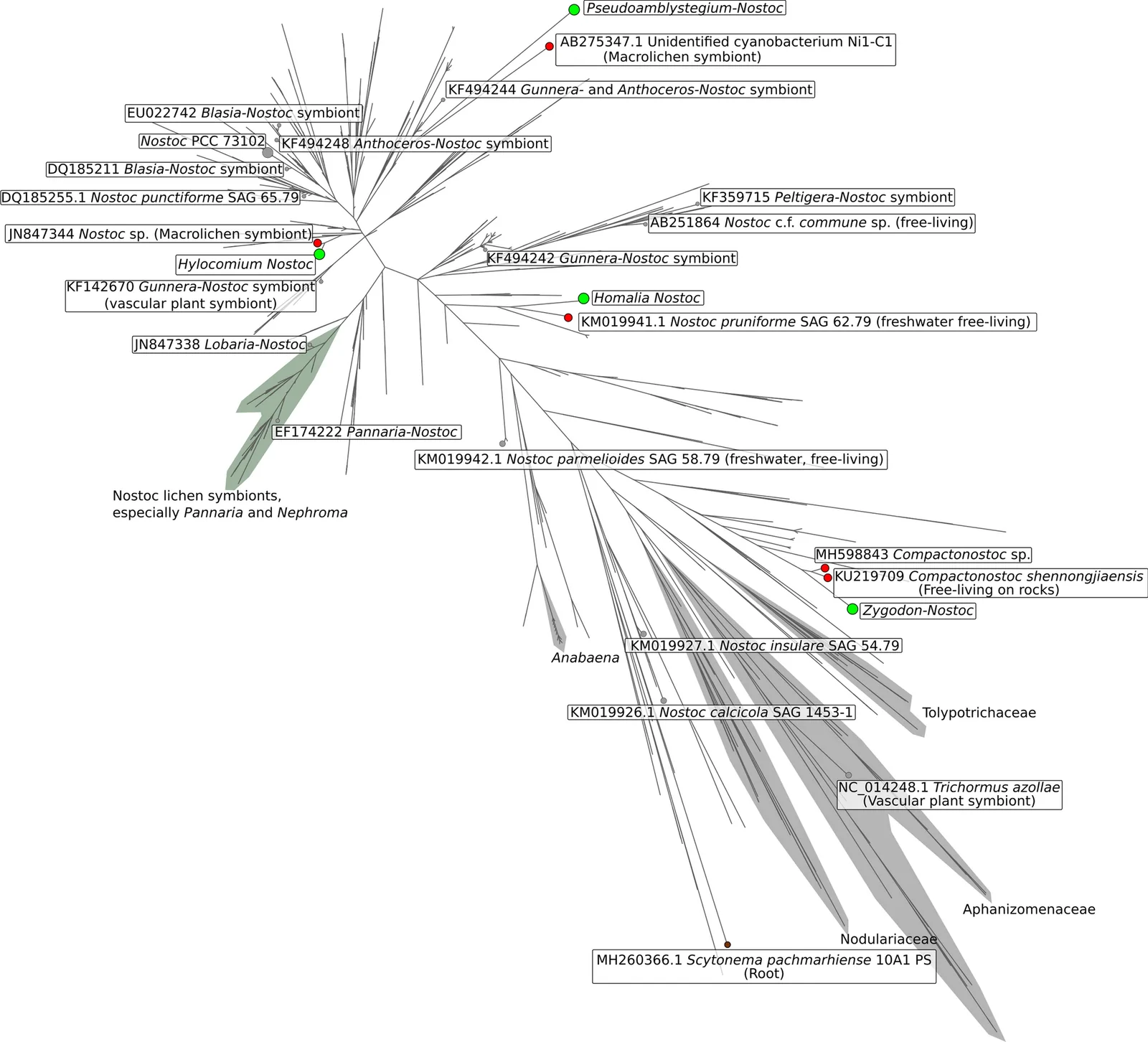
De novo 16 s Nostaceae phylogeny constructed from 856 full length 16S sequences from the Cyanoseq database (see methods), selected additional reference sequences, and four 16S consensus sequences from moss-associated Nostoc-like organisms from the present study (green circles). Nearest neighbors to moss-associated Nostoc-like organisms are shown by smaller red circles, and selected additional reference sequences are labeled by gray circles

Full-length 16S consensus sequences were determined for all 4 successfully cultured *Nostoc*- like organisms (Fig. 9, green circles). Only one consensus *Nostocaceae* 16S sequence was detected for each of the cultures. In the case of *Pseudoamblystegium,* 16S sequence data were overwhelmingly non-*Nostocaceae* cyanobacterial reads that were not identifiable beyond phylum-level (Cyanobacteriota), likely due to co-cultured cyanobacteria (see above). However some high-quality 16S reads remained for identification of the *Nostoc*-like organism originally observed for this sample.

Observed *Nostoc*-like moss symbionts were placed throughout *Nostoc*aceae (Fig. 9, Table 2), though nearest neighbors were in several cases previously known symbionts: in *Pseudoamblystegium* and *Hylocomium* mosses, the moss-associated *Nostoc* were closely related to endosymbionts of macrolichen-symbionts (Miura and Yokota 2006; Olsson et al. 2012) and the vascular plant *Gunnera magellanica* (Fernández-Martínez et al. 2013). For *Zygodon* moss, the cyanobacterium we observed was most closely related to the recently described genus *Compactonostoc* (Cai et al. 2019). *C. shennongjiensis* was reported as free living on rocks, another secondary habitat known for *Zygodon* species. For the moss *Homalia trichomanoides*, the closest relative was the freshwater-dwelling *Nostoc pruniforme,* as per Experimental Phycology and Culture Collection of Algae (SAG) strain 62.79, and other free-living *Nostoc* strains. Closest matches to other 16S sequences from *Nostoc*aceae are shown in Table 2.

**Table 2:** Closest sister taxa to observed Nostoc-like organisms observed from mosses in this study, based on our de novo 16S tree of Nostocaceae (Fig. 9)

*Nostoc*-like organism sequence ID (Moss host)	Nearest neighbor sequence ID and description	Blastn alignment scores (identities, gaps)
PX869778 (*Hylocomium*)	JN847344 *Leptogium*-associated *Nostoc* sp. (macrolichen symbiont)	1395/1405 (99%), 8 (1%)
PX856665 (*Homalia*)	KM019941 *Nostoc pruniforme* SAG 62.79 (freshwater, free-living)	1353/1383 (98%), 6 (0%)
PX856664 (*Pseudoamblystegium*)	AB275347 Unidentified cyanobacterium Ni1-C1 (unidentified-macrolichen symbiont), classified as likely *Nostoc* by Cyanoseq	677/710 (95%), 11 (2%)
PX856666 (*Zygodon*)	MH598843 *Compactonostoc shennongjiaensis* (Free-living on rocks)	1150/1168 (98%), 8 (1%)
	KU219709 “*Nostoc*” sp., unpublished, classified as likely *Compactonostoc* by Cyanoseq	904/915 (99%), 6 (1%)

When a close match is available, only one taxa is listed. When a close match is not obvious, the two nearest neighbor taxa are listed

## Discussion

Here we have documented additional instances of symbioses of *Nostoc* and *Nostoc-*like organisms with several mosses from the region of middle and upper Franconia, Germany, utilizing a microscopy-, culture-, and barcoding-based approach. Our survey therefore expands the known range of these symbioses further into southeastern Germany. We have also expanded the range of known moss hosts for *Nostoc* to include two non-feather mosses, in the family Orthotricaceae (order Orthotrichales), represented here by *Zygodon* and *Orthotrichum.* Additional new host moss families within *Hypnales* were also documented: *Pseudoamblystegium subtile* (Amblystegiaceae) and *Homalia trichomanes (*Neckeraceae).

The finding of *Nostoc* symbiosis in forest-floor *Hylocomium splendens* deeper into central Europe was predicted, since the well-known host moss species *H. splendens* and *Pleurozium schreberi* are very common in the region (supp. fig. 6) (Meinunger et al. 2007). Nevertheless, the extension of the geographical range of this well-known symbiosis further emphasizes their likely importance to montane forest ecosystems. The contribution of these symbioses to the overall nitrogen input into forest ecosystems of our region of Central Europe may be significant, given the high rates of nitrogen fixation by moss-microbiomes in observed in German forests (Groß et al. (2024), the documented importance of moss-hosted cyanobacterial nitrogen in boreal forests and other ecosystems (discussed above), and the abundance of these mosses in our region.

There are obvious disadvantages to a culture-based approach, primarily that presumably many *Nostoc*-like organisms were not successfully cultured. Cultures that were successful were extremely slow-growing, and were in one case not mono-cyanobacterial. Generally, target *Nostoc*-like organisms were often outcompeted in culture by other photoautotrophs. *Nostoc*-like organisms exist in many other life stages besides multicellular colony stages, including single-cell and single-trichome life stages (Mollenhauer 1986). *Nostoc* present on mosses in these life stages probably often escape detection by standard light microscopy. Our PCR-tests of moss tissue with cyanobacterial *nifH* genes (supp. fig. 4) support this, indicating that cyanobacteria are likely present in most aerial tissues of *Hylocomium* even when not detectable with microscopy. As such, the organisms cultured here likely greatly under-represents the diversity of *Nostoc*-like organisms in mosses in our region, and even more so the diversity of nitrogen-fixing cyanobacteria and nitrogen fixers generally in these mosses.

Despite the limitations of our culture- and microscopy-based approach, we documented new moss hosts for *Nostoc*-like organisms. Particularly of interest are host species *Zygodon dentatus* and *Orthotrichum lyellii*, both of Orthotricaceae and both of which fall outside of the category of feather mosses (order Hypnales). Feather mosses and *Sphagnum* are traditionally understood to be the hosts for moss-*Nostoc* symbioses, but we show here that such symbioses may be occurring in other distantly-related moss groups.

The present study did not include conduct systematic sampling for *Nostoc*-like organisms. This limits our ability to draw conclusions about the abundance and spatial distribution of these symbioses in the forest soil or canopy, and our ability to estimate the extent of their importance in nutrient cycling in central European forests. Some of the mosses examined, especially epiphytic mosses such as *Zygodon*, are rare in the study range, making quantification additionally difficult. Future scientific efforts to understand the importance of these symbioses will require spatially explicate sampling and significantly more replication.

Despite this limitation, it is intriguing that two new host mosses (*Zygodon dentatus* and *Orthotrichum lyellii*) were arboreal mosses. Four out of five moss hosts in which we observed host *Nostoc*-like organisms were epiphytic on tree bark (Table 2). This is perhaps supportive of the hypothesized importance of this symbiosis in forest canopies.

(Lindo and Whiteley 2011), and we hypothesize that N-fixing cyanobacterial symbionts may provide an important mechanism for allowing moss communities to thrive and compete in the very challenging microclimate of bark. Cyanobacteria in arboreal mosses have been observed previously, i.e. in the coastal temperate rain forest of North America by Lindo and Whiteley (2011) and in a subtropical montane cloud forest in southwestern China for the species *Homaliodendron montagneanum*, *H. scalpellifolium* and *Thuidium cymbifolium* (Fan et al. 2022). Physically smaller and more ephemeral mosses such as *Zygodon* spp. or *Pseudoamblystegium* spp., as well as initial or young stages of larger mosses like *Orthotrichum*, may especially benefit from this symbiosis, as smaller mosses may not be able to access biologically available nitrogen from atmospheric deposition or stemflow as well as larger mat-forming mosses. If this is true, we would predict symbiosis with cyanobacteria to be especially prevalent on the smaller species as hosts, as we observed for *Zygodon*, *Pseudoamblystegium subtile* and *Homalia*, all of which are comparatively physically small mosses of their families (Frahm and Frey 2004). Whereas the genus *Orthotrichum* is widespread and common in our region, the genus *Zygodon* is rare in central Europe (supp. fig. 6) (Meinunger et al. 2007) and occurs ephemerally in very scattered mats at sites with a high humidity. *Zygodon* has comparatively small gametophytes (Frahm and Frey 2004) and may be at a competitive disadvantage compared to larger, co-occuring species of *Orthotrichum*, and the moss-*Nostoc* symbioses may be especially important for these mosses for continued survival. Additionally, *Zygodon* spp. and *Orthotrichum lyellii* produce large amounts of multicellular gemmae for dispersal (Frahm and Frey 2004). This continual production of gemmae may create additional demand on these mosses for organic nitrogen, and therefore symbioses as we observe here.

We found no clear phylogenetic signal among the symbiotic *Nostoc*-organisms for which the 16S rRNA gene were successfully sequenced—symbionts were placed throughout the Nostocaceae family (Fig. 9, Table 2). Nearest matches to other *Nostoc* full length 16S sequences were from various ecological niches, both symbiotic and free-living. Additionally, according to 16S sequence data, our *Nostoc*-like organism isolated from *Zygodon* is most closely related to the recently-described genus *Compactonostoc* (Cai et al. 2019). This genus of *Nostoc*-like organisms has to-date been observed from freshwater free-living states (Cai et al. 2019; Caro-Borrero et al. 2024), but our observation implies that members of *Compactonostoc* may be capable of plant-symbiosis.

Another benefit of our culture-based approach is that we are able to link taxonomic identity ( 16S rRNA sequences) to a functional gene (*nifH*) within single organisms. This has enabled the observation of possible alternate nitrogenases (3 distinct *nifH* copies) in the genomes of 2 of our moss-associated *Nostoc*-like organisms. Alternative nitrogenases have been previously observed from members of Nostocaceae (Thiel 1993; Thiel et al. 1995), and are thought to perform supplementary or complementary roles, allowing diazotrophy in different oxygen levels, or the use of alternative metal co-factors (vanadium or iron-only) in place of molybdenum (Zehr et al. 2003). It is worth noting that our only terrestrial moss (*Hylocomium splendens*) did not appear to host *Nostoc* with alternate nitrogenases, only our arboreal *Nostoc* were observed to have alternate *nifH* versions. The dynamic habitat of the forest canopy may select for homologs of nitrogenase genes with different environmental optima and nutrient requirements, enabling diazotrophic cyanobacteria to fix nitrogen and continue growth under a wider range of conditions.

The possibility that moss-associated cyanobacteria host multiple nitrogenases has implications for the study of moss-associated *Nostoc* and their ecological services. For example, Warshan et al. (2016) and Arróniz-Crespo (2022) attribute relative importance in terms of biological nitrogen fixation to different cyanobacterial groups in boreal forests, based on differential abundances of sequences falling into *nifH* clades defined by Ininbergs et al. (2011). However, we show here that great care should be taken when assigning taxonomy using these *nifH* clusters. Warshan et al. (2016) and Arróniz-Crespo (2022) suggest *Stigonema* as the most important group contributing to fixation of nitrogen in boreal forest systems based on high relative abundance or high levels of expression of *nifH* genes aligned to the “*Stigonema* cluster” of *nifH*, sensu Ininbergs et al. (2011). Our results indicate that the *Nostoc* strain cultured from *Homalia trichomanoides*, closely related to *Nostoc pruniforme* SAG 62.79 (Fig. 9), has a version of *nifH* that falls within this “*Stigonema*” cluster, but also hosts two other *nifH* genes from other clusters sensu Ininbergs et al. (2011) (Fig. 8 and Supp. fig. 5).

We also observed the possible endophytic colonization of moss stems by cyanobacteria, based on the presence of autofluorescing cells in moss tissue following epiphyte-reduction, and amplification of *nifH* in mosses where no *Nostoc-*like colonies were observable via microscopy (Fig. 7 and supp. fig. 4). However, PCR protocols used for detection of *nifH* resulted in over-amplification and indicated the presence of abundant *NifH* homologs from host moss tissue, reducing confidence in interpretation of PCR results. It is also very possible that the epiphyte reduction protocol utilized here was insufficient to remove all epiphytes, and that in our microscopy we are observing remnant epiphytic microbiota or even plant tissue autofluorescence.

Endophytism by *Nostoc* in plants has been observed previously. For example, *Nostoc punctiforme* PCC 73102 is endophytically competent and capable of endophytically colonizing roots of rice (Álvarez et al. 2020), and is a close relative to our *Hylocomium splendens-*associated *Nostoc-*like organism. Among mosses, cyanobacterial endophytes are only previously known in *Sphagnum*, which hosts *Nostoc* and other bacteria in specialized structures known as hyalocytes, or dead, hyaline leaf cells (Carrell et al. 2022). Moss leaflet lamina are typically only one cell thick (Roberts et al. 2012), precluding abundant endophytic colonization of leaves by microbes that is commonly observed in vascular plants (Bodenhausen et al. 2013; Rodriguez et al. 2009). Stems (‘caulids’) and rhizoids of mosses are multicellular in cross-section, and are therefore presumably the site of colonization of much of the extensively documented, diverse non-cyanobacterial endophytes observed in mosses (Tveit et al. 2020; U’Ren et al. 2010). If the observed cells are indeed cyanobacteria occupying the stem tissue of mosses, it points to an abundant symbiosis. We found large epiphytic colonies of *Nostoc*-like organisms relatively rarely in our mosses, so the possibility exists that much of the relatively high rates of nitrogen fixation by cyanobacteria among mosses in our region as noted by Groß et al. (2024) may be achieved by potential endophytic symbionts instead. Endophytic colonization by *Nostoc* and other cyanobacteria is well documented in other plant- and algal-symbiotic systems, including in *Sphagnum* spp., and hornwort and liverwort hosts (reviewed by Adams and Duggan (2008)), albeit in protective plant compartments such as hyalocytes, auricles, or slime-cavities. Further experimental work is needed to determine the epiphytic/endophytic location of these cells, to confirm their taxonomy as cyanobacterial cells, and to explore their potential for nitrogen fixation, if any.

## Conclusion

Here we extend the known geographical range of the classical feather moss *Hylocomium*/*Nostoc* symbiosis further into the region of central Europe, highlighting the importance of moss layers for nitrogen cycling in montane forests of central Europe. We expand the known host range of to include additional non-feather mosses (*Orthotrichum* and *Zygodon*), as well as to other feather mosses not previously observed to host *Nostoc* symbionts (*Pseudoamblyestegium, Homalium*). We note that multiple versions of *nifH* can be found within some *Nostoc-*like organisms, which may denote adaptation to the dynamic environment of the forest canopy, but which also implies that great care be applied when attempting to identify these *Nostoc*aceae symbionts with *nifH* gene sequence data. We also report a potential site of high cyanbacterial colonization, namely the endophytic compartment of the “stem” (caulid) of mosses. If occurring, this implies an abundant, physically-protected symbiosis but not with *Nostoc,* or at least not in the large-colony stages.

Taken together, these results help to bridge the gap between two commonly observed forest moss symbioses: (1) few-to-few, feather moss/*Nostoc* symbioses, and (2) many-to-many moss/cyanobacterial-community symbioses. From our survey, the former appear to occur in our region but relatively rarely. Based on other reports (cited above), the latter class of broader cyanobacterial symbioses is apparently ubiquitous in mosses where nitrogen is limiting, including in our region.

## Supplementary Information

Below is the link to the electronic supplementary material.

Supplementary file1 (PNG 1020 KB) **Supplementary figure 1**. Laboratory pipeline, with moss host species sampled at each step.

Supplementary file2 (PNG 3721 KB) **Supplementary figure 2**. Bioinformatic pipeline for 16S and *nifH* sequence data.

Supplementary file3 (PNG 6421 KB) **Supplementary figure 3**. Unrooted trees of filtered raw reads from *nifH* sequence data, for *Nostoc-*like organisms isolated from *Zygodon, Homalia, *and *Hylocomium *host mosses. Reads as of tightly clustered subtrees (clades) were used as the basis for detection and description of alternate *nifH *versions. Grey sequence box indicates a reference *nifH* sequence from the genome of *Nostoc punctiforme* PC.

Supplementary file4 (PNG 146 KB) **Supplementary figure 4**. Electrophoresis gel ol PCR of nifH region, using cyanobacterial specific nifH primers on whole community DNA extracted from Hylocomium splendens. Bands above 700 bp in size are presumed to be chimeric in origin due to overamplification. Bands in the region of 500 bp are hypothesized to be embryophyta homologs of *nifH*, due to their presence in epiphyte reduced, urban *Brachythecium* moss and epiphyte reduced Solidago leaves, and their absence in axenic *T. variabilis* culture. Amplicons of size ~400 are presumed to be the intended the target of PCR reaction, cyanobacterial nifH, indicating the possible presence of nitrogen-fixing cyanobacteria in these moss tissues.

Supplementary file5 (PNG 1997 KB) **Supplementary figure 5**. *nifH *gene phylogeny of select members of Nostoceae. Tree is constructed from de novo alignment of *nifH *sequences detected in this study from Nostoc-like organisms (green) and reference sequences from Ininbergs et al. (2011) and Kubota et al. (2023). *Zygodon*- and *Homalia*-associated *Nostoc*-like organisms both hosted multiple distinct copies of *nifH*. Branch lengths are proportionate to expected number of nucleotide substitutions per site. Branch support values are Shimodaira-Hasegawa (SH) test values, ranging from 0 (low confidence) to 1 (high-confidence). Branch supports lower than 0.8 are multifurcated.

Supplementary file6 (PNG 10663 KB) **Supplemental figure 6**. Known species distributions of all moss host species in the state of Bavaria, according to Die Moose Bayerns website (https://bayern.moose-deutschland.de/ )

## Data Availability

Code and supporting files are available at the associated github.com repo in the folder "suppData", at the following link: <https://github.com/danchurch/scalingUpByLookingCloser_Feulner_etal/tree/main/suppData>. Demultiplexed, raw Nanopore reads are available at NCBI SRA, from the bioproject PRJNA1401537. 16 s rRNA and *nifH* consensus sequences are available at the NCBI Nucleotide Database. GenBank accession numbers for 16S rRNA consensus sequences are: PX869778, PX856665, PX856666, PX856664. GenBank accession numbers for nifH consensus sequences are PX892065, PX892066—PX892068, PX892069—PX892071.
